# The cell wall integrity pathway kinase Mpk1 regulates extracellular vesicle production and apoptotic-like vesicle release in *Cryptococcus neoformans*

**DOI:** 10.1128/mbio.02302-26

**Published:** 2026-10-23

**Authors:** Cassandre Deculty, Patricia Flamant, Frédérique Moyrand, Pierre-Henri Commere, Sophie Novault, Magdalena Gil, Rayen Elj, Mariette Matondo, Guilhem Janbon, Isabelle Mouyna

**Affiliations:** 1 RNA and Extracellular Vesicles Unit, Department of Mycology, Institut Pasteur, Université Paris Cité, Inserm U1359 555089, Paris, France; 2 Platform Cytometry and Biomarkers, Centre de Ressources et Recherches Technologiques (C2RT), Institut Pasteur, Université Paris Cité 555089, Paris, France; 3 Plateforme Protéomique, Unité de Spectrométrie de Masse pour la Biologie, Centre de Ressources et Recherches Technologiques (C2RT), Institut Pasteur, Université Paris Cité, CNRS UMR 2000 555089, Paris, France; Georgia Institute of Technology, Atlanta, Georgia, USA

**Keywords:** *Cryptococcus neoformans*, extracellular vesicles, apoptosis, cell wall integrity pathway

## Abstract

Extracellular vesicles (EVs) are important mediators of fungal physiology and host interactions, yet the mechanisms governing their production remain poorly understood. In *Cryptococcus neoformans*, EV production is tightly regulated by environmental conditions, suggesting the existence of dedicated signaling pathways. Here, we screened a kinase deletion library to identify regulators of EV production and found that the cell wall integrity (CWI) pathway acts as a major negative regulator of EV production. Deletion of the CWI kinases *MPK1*, *MKK2*, or *BCK1* increased EV release. Unexpectedly, the size of EVs produced by CWI mutants differed markedly from wild type. Accordingly, cryo-electron microscopy revealed the accumulation of larger vesicles with altered size distributions in the *mpk1*Δ mutant. These EVs lacked the characteristic mannoprotein-based fibrillar surface decoration observed on most wild-type vesicles, exhibited reduced association with capsular polysaccharide, and contained substantially less protein. Quantitative proteomic analysis demonstrated extensive remodeling of the EV proteome, with depletion of glycosylphosphatidylinositol (GPI)-anchored proteins, and enrichment of ribosomal proteins and histones. Finally, *mpk1*Δ EVs displayed several hallmarks of mammalian apoptotic EVs, including phosphatidylserine exposure, and were produced under conditions associated with reduced cell viability. Together, these findings identify the CWI pathway as a central regulator of fungal EV production and provide evidence that *C. neoformans* produces distinct EV populations through genetically regulated pathways. Our results provide evidence for an apoptotic-like EV population in fungi and reveal an unexpected link between cell wall integrity signaling, cell death, and EV diversity.

**IMPORTANCE:**

Fungal extracellular vesicles (EVs) contribute to virulence, antifungal resistance, and host-pathogen interactions, yet the mechanisms governing their production remain poorly understood. Here, we identify the cell wall integrity signaling pathway as a major regulator of EV production in the human fungal pathogen *Cryptococcus neoformans*. We further show that disruption of one of the major kinases of this pathway induces the production of a distinct population of EVs with structural and molecular features resembling mammalian apoptotic EVs. These findings demonstrate that fungi produce multiple EV populations through genetically regulated pathways rather than a single constitutive mechanism. By establishing the existence of apoptotic-like EVs in fungi, our work provides new insight into fungal EV production and lays the foundation for investigating how different EV populations contribute to fungal physiology, pathogenesis, and antifungal resistance.

## INTRODUCTION

Extracellular vesicles (EVs) are particles surrounded by a lipid bilayer that are released by cells into the extracellular environment. They have been extensively studied in mammalian systems, where they mediate intercellular communication, immune modulation, and disease progression (1). Fungal EVs were first described in the pathogenic yeast *Cryptococcus neoformans* and have since been identified across a wide range of fungal species, including yeasts, molds, and filamentous fungi (2–5). Over the past decade, the study of fungal EVs has gained increasing attention, revealing a previously underappreciated mechanism by which fungi interact with their environment and host organisms. Fungal EVs are produced during infection (6) and contribute to host–pathogen interactions (7–11), antifungal resistance, regulation of virulence factor expression, and biofilm formation (12–14). Their cargo includes lipids, polysaccharides, pigments, proteins, metabolites, and nucleic acids, although the functions of many of these molecules remain poorly understood (5). Our recent structural analysis of *C. neoformans* EVs revealed extensive diversity in size, shape, and architecture, with most EVs being decorated with fibrillar mannoproteins and coated with capsular polysaccharides (15).

Despite these advances in defining fungal EV composition and structure, the mechanisms governing EV production remain largely unknown. In mammalian cells, EVs are generated through multiple pathways that give rise to distinct EV populations, including exosomes, microvesicles, and apoptotic vesicles (16). Because EV origin is often difficult to establish experimentally, the International Society for Extracellular Vesicles currently recommends classifying EVs according to their size (small, medium, and large EVs), unless their biogenesis has been demonstrated (17). This recommendation reflects the absence of universal markers that reliably distinguish EV classes and the fact that vesicle size alone does not predict cellular origin (18, 19).

Evidence suggests that fungal EVs are similarly heterogeneous. Distinct EV size populations have been reported, and their relative abundance can be differentially regulated (15, 20), raising the possibility that fungi produce multiple EV types through distinct biogenetic pathways. By analogy with mammalian cells, some fungal EVs may originate from the endosomal pathway through multivesicular bodies, although evidence remains largely limited to microscopy studies, such as those performed in the plant pathogen *Colletotrichum higginsianum* (5, 9). Other EVs may arise by direct budding from the plasma membrane. Consistent with this hypothesis, mutations affecting components of the ESCRT machinery, which participates in both exosome and microvesicle biogenesis in mammals, reduce EV production in *Candida albicans* (12). In addition, apoptosis-like processes have been described in fungi (21), although apoptotic EVs (ApoEVs) have not yet been identified. Thus, the existence and relative contribution of distinct fungal EV biogenesis pathways remain unresolved. Likewise, the mechanism by which EVs traverse the fungal cell wall remains unknown despite several proposed models (5).

We previously demonstrated that EV production in *C. neoformans* is tightly regulated by environmental conditions, suggesting that dedicated signaling pathways control EV production (14). Such pathways typically consist of receptors, signaling intermediates, and downstream effectors that coordinate cellular responses to environmental stimuli (22). In an earlier genetic screen, we identified four transcription factors—Hap2, Gat5, Liv4, and Bzp2—that positively regulate EV production in *C. neoformans*. Deletion of any of these regulators markedly impaired EV secretion and simultaneously altered susceptibility to fluconazole, suggesting a functional link between EV production and antifungal resistance (14).

Here, we extended this approach by screening a kinase mutant library to identify signaling pathways that regulate EV production. We identified the cell wall integrity (CWI) pathway as a major negative regulator of EV production in *C. neoformans*. Unexpectedly, nano-flow cytometry, cryo-electron microscopy (cryo-EM), proteomics, and genetic analyses demonstrated that EVs produced by the *mpk1*Δ mutant differ markedly from wild-type EVs in both morphology and molecular composition. These vesicles display features reminiscent of mammalian apoptotic vesicles, providing evidence that fungi can generate distinct EV populations through different biogenetic mechanisms.

## RESULTS

### CWI pathway negatively regulates EV production in *C. neoformans*

**Fig 1:**
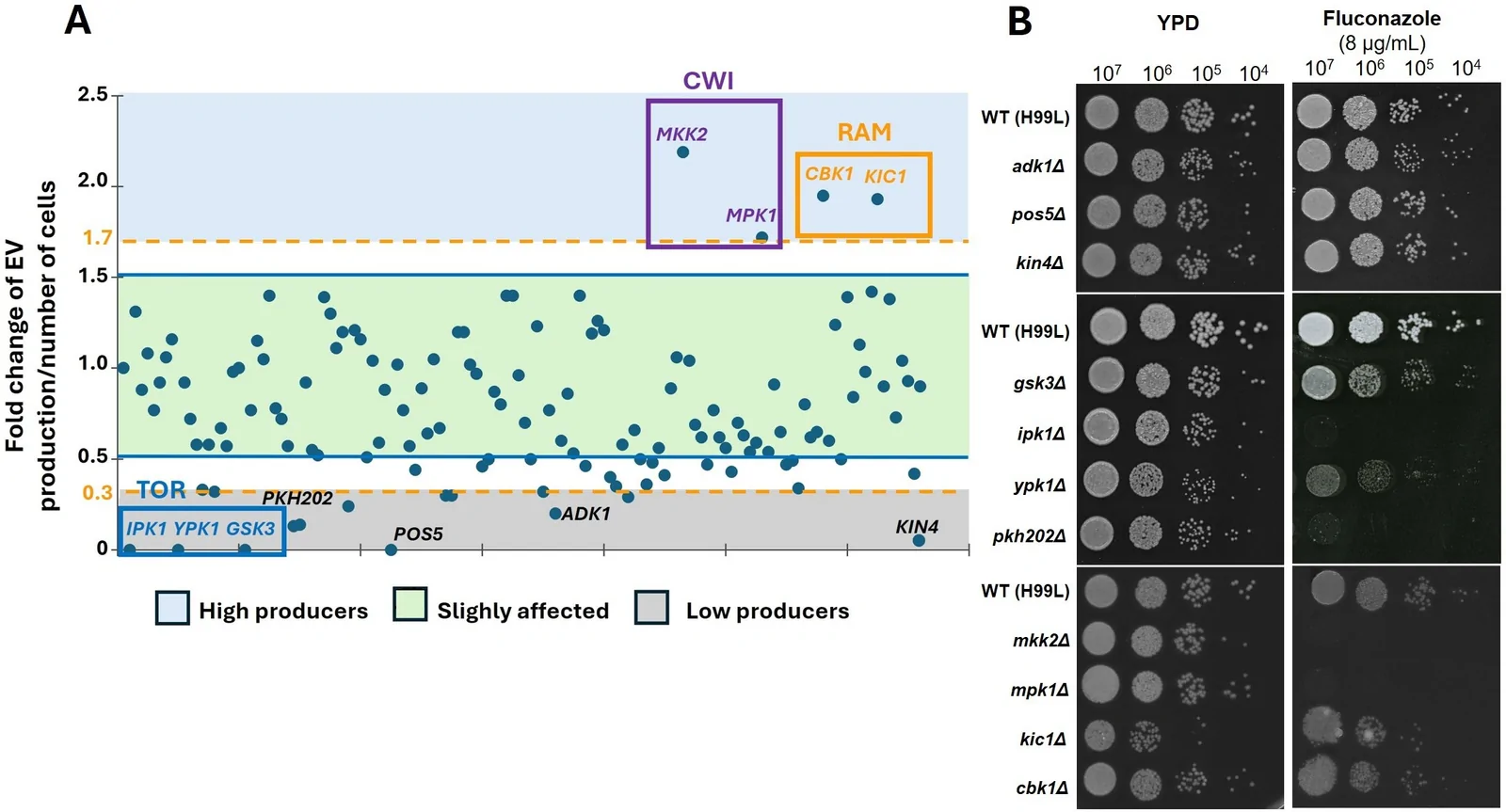
Screening of a kinase mutant collection identifies kinases regulating EV production and fluconazole susceptibility. (**A**) Identification of kinases regulating EV production in *C. neoformans*. Kinase mutant strains were grown on synthetic dextrose (SD) medium, and EVs were isolated using a rapid extraction protocol (24). EV production was quantified by measuring the sterol content of EV preparations and normalized to the number of cells recovered from each plate. Mutants producing >1.70-fold the EV levels of the wild-type strain were classified as high EV producers, whereas mutants producing <25% of wild-type EV levels were classified as low EV producers. (**B**) Kinase mutants with altered EV production exhibit altered susceptibility to fluconazole. Serial dilutions of the 11 kinase mutants impaired in EV production were spotted onto yeast extract-peptone-dextrose (YPD) medium containing fluconazole (8 µg/mL). Plates were incubated for 48 h at 30°C before imaging.

To identify signaling pathways that regulate EV production in *C. neoformans*, we screened a collection of 130 kinase deletion mutants (23). EV production was quantified after growth on solid medium using the assay previously developed in our laboratory (15, 24). Seven mutants produced less than 25% of the amount of EVs released by the wild-type strain, whereas four mutants produced more than 1.70-fold as many EVs (Fig. 1A; Table 1; Table S1).

**TABLE 1:** List of the eleven kinase mutants involved in the regulation of EV production in *C. neoformans^a^*

Type	Genes mutated	EV ratio/WT (H99L)	Functional role
Low EV producers	*IPK1*	0	Involved in inositol polyphosphate biosynthesis
	*YPK1*	0	Involved in the Target Of Rapamycin (TOR) pathway
	*GSK3*	0	
	*PKH202*	0.14	Involved in sphingolipid pathway
	*ADK1*	0.2	Involved in cell energetic homeostasis by interconverting adenine nucleotides
	*POS5*	0	Involved in mitochondrial NADPH biosynthesis and important for the resistance to oxidative stress and mitochondrial DNA stability
	*KIN4*	0.02	Involved in the spindle position checkpoint: delays the cell cycle progression in case of spindle pole bodies position issue
High EV producers	*MKK2*	2.19	Involved in the CWI pathway: cell wall biogenesis and remodeling
	*MPK1*	1.72	
	*KIC1*	1.93	Involved in the RAM pathway: cell polarity
	*CBK1*	1.95	

^*a*^ The values shown represent the mean of at least three independent experiments performed for each of the two independent deletion mutants in the kinase mutant library.

Consistent with our previous observation that EV production and fluconazole susceptibility are co-regulated (14), 8 out of 11 kinase mutants with altered EV production also displayed altered fluconazole susceptibility (Fig. 1B), further supporting a link between EV production and antifungal resistance.

Although the identification of multiple regulators of EV production provides promising avenues for future investigation, some of which are currently underway in our laboratory, we focused on the two EV high producer mutants, *mpk1*Δ and *mkk2*Δ, which lack components of the MAPK module of the CWI pathway (25). This pathway is required for adaptation to cell wall stress (26) and intersects with several signaling pathways represented among the mutants identified in our screen. For example, the remaining high-EV mutants, *cbk1*Δ and *kic1*Δ, affect components of the RAM pathway, which extensively cross-talks with the CWI pathway in *C. neoformans* and *Saccharomyces cerevisiae* (27–29). Conversely, two low-EV mutants (*ypk1*Δ and *gsk3*Δ) affect TOR signaling, which negatively regulates the CWI pathway in these yeasts (30, 31). Among the four kinases comprising the CWI MAPK module (Mpk1, Mpk2, Mkk2, and Bck1) (25), only *MPK1* and *MKK2* were identified in our screen. Strangely enough, the *bck1*Δ mutant (listed as *mkh1*Δ in Table S1) did not display any EV production phenotype. However, this phenotype was tested only once, and the purity of the strain was not verified. Finally, the *MPK2* deletion had no effect on EV production, consistent with its distinct biological function (32) (Table S1).

Because the kinase collection was generated in the H99L (33) background, whereas our laboratory routinely uses KN99α for genetic analyses (34), we re-evaluated the phenotypes of the CWI mutants in the KN99α background. We obtained *mpk1*Δ and *bck1*Δ (CNAG_04755) mutant strains from the Madhani deletion collection (35) and generated an *MKK2* (CNAG_02357) deletion strain, which is absent in this genome-wide mutant collection. Finally, we constructed the *mpk1*Δ*::MPK1* and *mkk2*Δ*::MKK2* complemented strains by integrating the corresponding wild-type genes at the Safe Haven 1 locus (36). All three mutants displayed increased susceptibility to fluconazole (Fig. S1), Calcofluor White, Congo Red, SDS, and elevated temperature (Fig. S1). They also exhibited altered Calcofluor White staining and reduced capsule size, in agreement with previous reports (23, 32, 37, 38). All phenotypes were restored in the complemented strains (Fig. S1).

**Fig 2:**
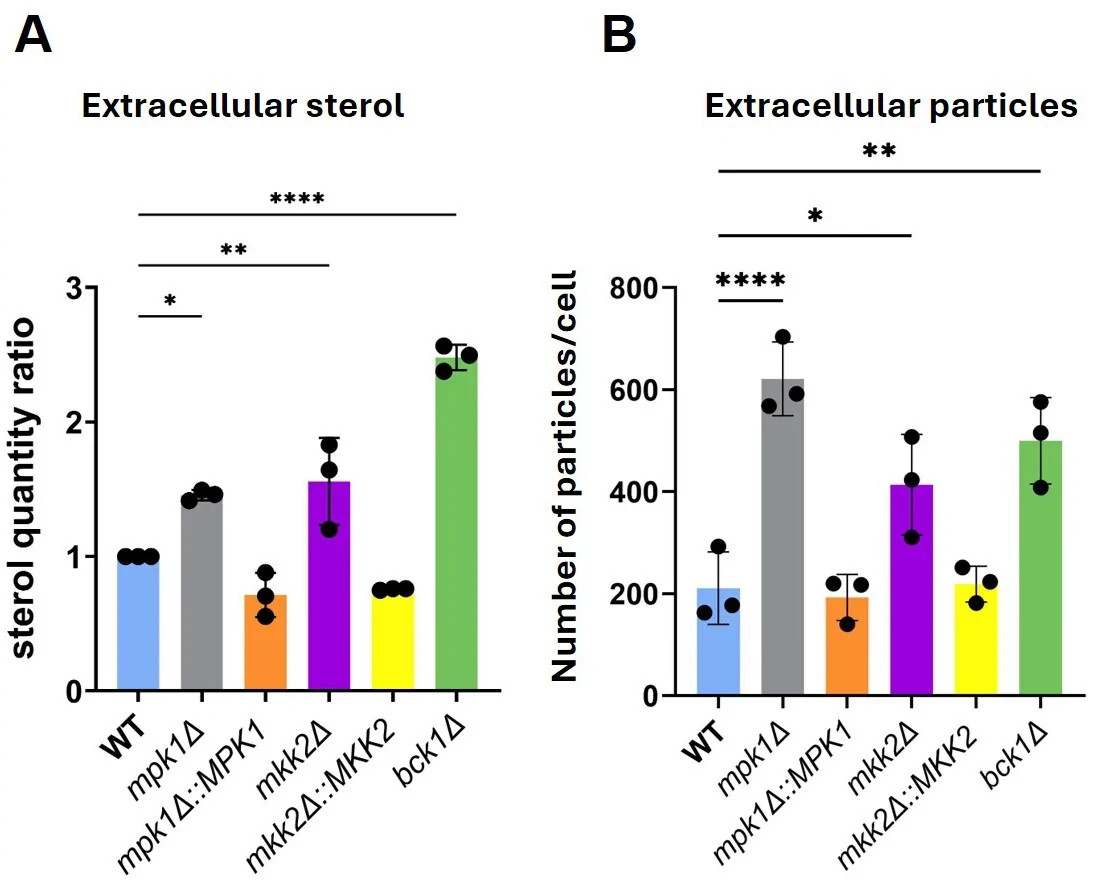
Deletion of CWI genes is associated with altered EV production in *C. neoformans*. (**A**) EV production by CWI mutants and their complemented strains after 22 h of culture at 30°C on synthetic dextrose medium, evaluated by measuring total sterol content in EV samples. (**B**) EV production by CWI mutants and their complemented strains was assessed by nanoflow cytometry (NanoFCM). EVs were isolated using a rapid extraction protocol (24). Statistical analyses were performed using a one-way analysis of variance followed by Dunnett’s multiple comparisons test. Error bars represent the mean of three biological replicates ± SD. *P < 0.05, **P < 0.01, and ****P < 0.0001.

We also confirmed that deletion of *MPK1*, *MKK2*, or *BCK1* significantly increased EV production, whereas complementation restored WT levels (Fig. 2A and B). This phenotype was detected using both sterol quantification and nanoparticle flow cytometry and was independent of the EV isolation procedure (Fig. 2A and B) (Fig. S2). These data identify the CWI pathway as a negative regulator of EV production in *C. neoformans*.

### The CWI pathway regulates EV size

**Fig 3:**
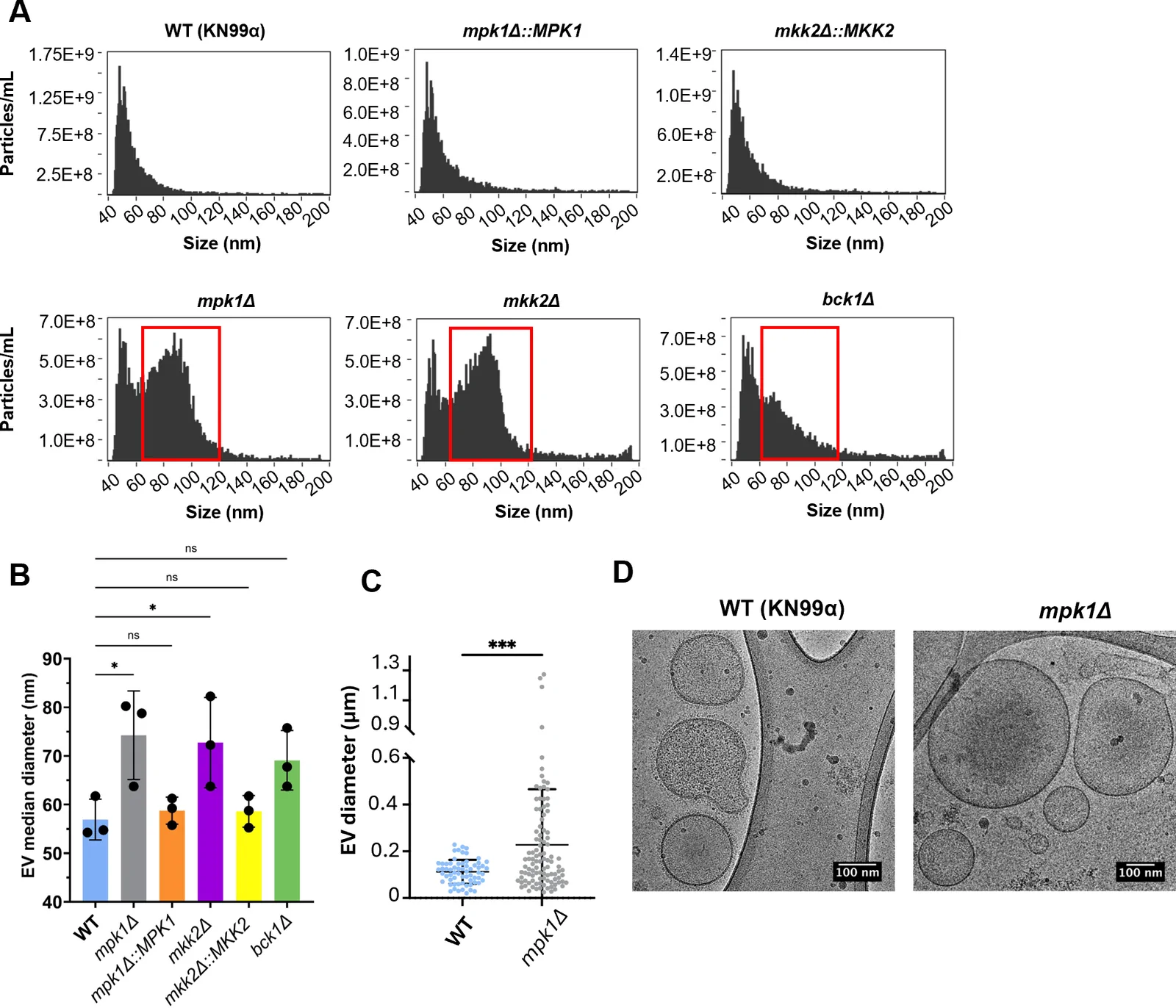
Analysis of EV size produced by CWI mutant strains. (**A**) EV size profiles obtained by NanoFCM for the wild type, complemented strains, and CWI mutant strains. Red squares indicate a second EV population present in the CWI mutants. (**B**) Median EV diameter as measured by NanoFCM. Statistical significance was determined by one-way analysis of variance followed by Dunnett’s multiple comparisons test. Error bars represent the mean ± SD of three biological replicates. ns, not significant; *P < 0.05. (**C**) Diameter of EVs from the WT and *mpk1*Δ strains as determined by analysis of cryogenic electron microscopy (cryo-EM) images. *n* = 64 EVs for WT and *n* = 103 EVs for *mpk1*Δ coming from a single EV preparation. Statistical significance was assessed using an unpaired two-tailed *t* test; ***P < 0.001. (**D**) Representative Cryo-EM images of EVs from WT and *mpk1*Δ mutant.

NanoFCM revealed an additional shoulder in the EV size distribution of all three CWI mutants that was absent from WT and complemented strains (Fig. 3A). Accordingly, CWI mutant EVs displayed larger median diameters (63.8–82.3 nm) than WT EVs (54.3–61.8 nm; Fig. 3B). Cryo-EM confirmed this phenotype in the *mpk1*Δ mutant, whose EVs were significantly larger than those of WT cells (Fig. 3C and D). Although cryo-EM is less efficient than cryo-electron tomography for detecting small vesicles (15) and underestimates the abundance of small vesicles relative to NanoFCM, both methods consistently demonstrated that disruption of the CWI pathway increases EV size.

### Disruption of *MPK1* alters EV structure

**Fig 4:**
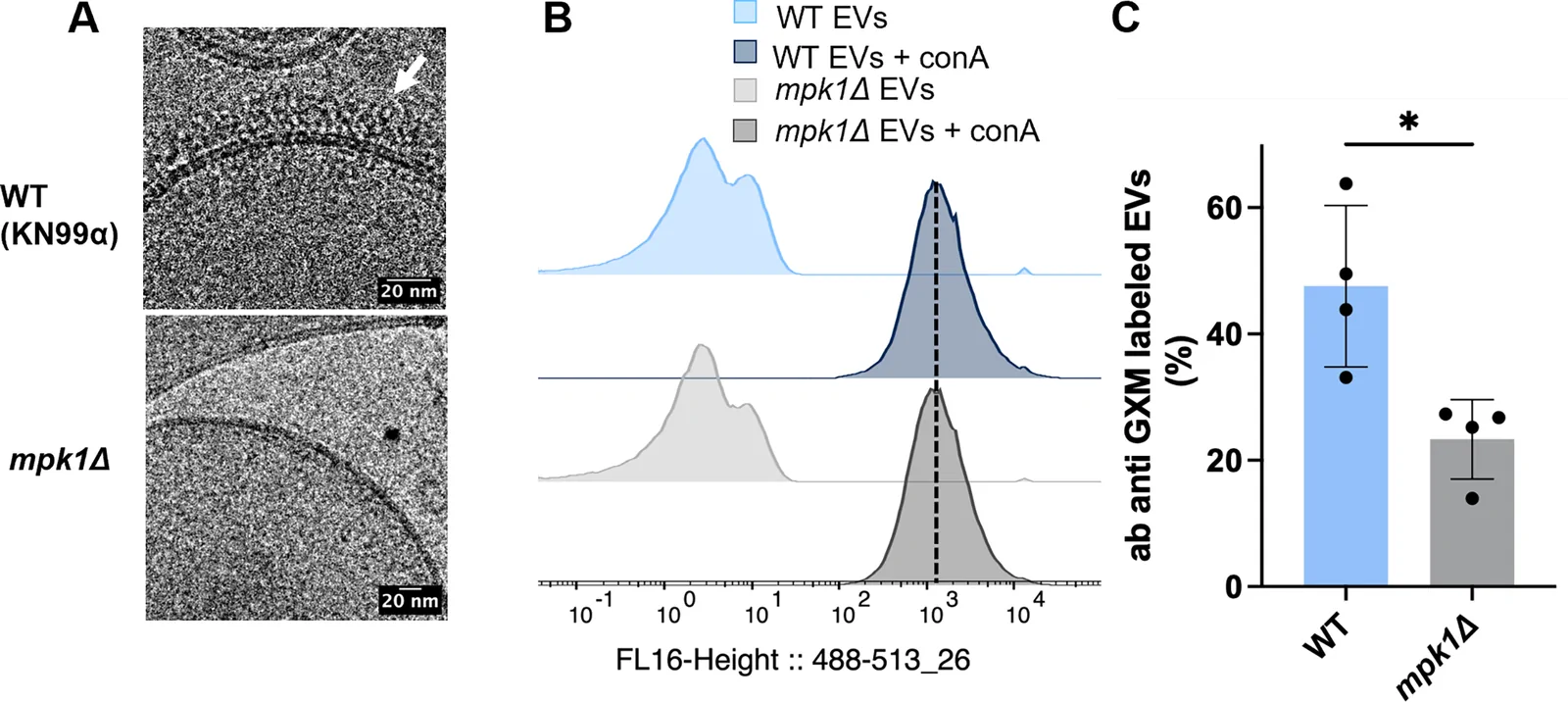
Deletion of *MPK1* alters EV structure (**A**) Representative cryo-electron microscopy images illustrating differences in fibrillar surface decorations of EVs produced by the wild-type and *mpk1*Δ strains. (**B**) Flow cytometry analysis of WT and *mpk1*Δ EVs labeled with concanavalin A (ConA)–Alexa Fluor 488. A total of 100,000 events were analyzed in the presence or absence of the lectin. (**C**) Flow cytometry analysis of WT and *mpk1*Δ EVs labeled with the monoclonal anti-GXM antibody 18B7. Data were acquired using a MoFlo Astrios flow cytometer (Beckman Coulter). Statistical significance was assessed using an unpaired two-tailed *t* test; *P < 0.05.

Consistent with our previous observation, cryo-EM analysis showed that approximately 60% of EVs produced by the wild-type strain displayed surface decoration. In contrast, only 8% of EVs produced by the *mpk1*Δ mutant exhibited detectable surface decoration (Fig. 4A). Surprisingly, ConA labeling detected similar proportions of mannose-positive vesicles in both strains (Fig. 4B), indicating that mannose-containing glycans remain associated with mutant EVs despite the loss of visible fibrillar structures. In contrast, and consistent with the reduced capsule size observed in CWI pathway mutants, nanoflow cytometry analysis of EVs labeled with an anti-capsular antibody revealed a marked reduction in the proportion of GXM-positive EVs in the *mpk1*Δ mutant (23.3%) compared to the parental strain (47.6%; Fig. 4C). Together, these data demonstrate that Mpk1 regulates EV surface architecture.

### EV protein composition is altered in *mpk1*Δ

**Fig 5:**
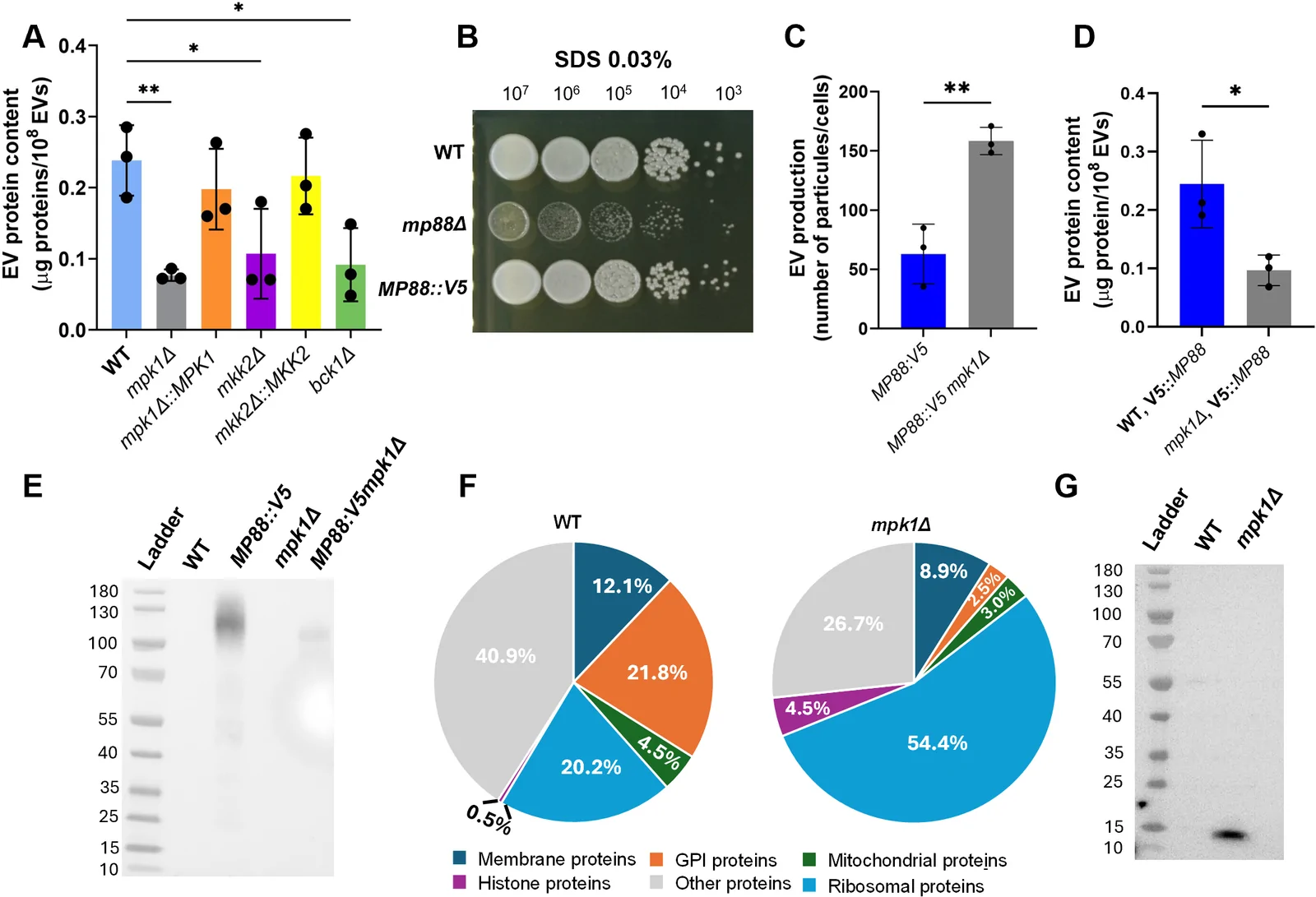
*MPK1* deletion affects the EV proteome. (**A**) Protein concentrations in each EV preparation were quantified using a Pierce BCA protein assay kit and normalized to the number of EVs. (**B**) Spot assay analysis confirms that *MP88::V5* is functional. Three microliters of 10-fold serial dilutions of wild-type (KN99α), *mp88*Δ, and *MP88::V5* cells were spotted onto yeast extract-peptone-dextrose (YPD) plates containing 0.03% (wt/vol) SDS. Plates were incubated at 30°C for 48 h before imaging. (**C**) Deletion of *MPK1* in the *MP88::V5* background is associated with increased EV production, as estimated by NanoFCM. (**D**) EVs produced by the *MP88::V5 mpk1*Δ mutant contain less protein than those produced by the *MP88::V5* strain. Protein concentrations in each EV preparation were quantified using a Pierce BCA Protein Assay Kit and normalized to the number of EVs. (**E**) Mp88 is less abundant in EVs isolated from the *mpk1*Δ mutant. Western blot analysis was performed on EV protein extracts from WT, *mpk1*Δ, *MP88::V5*, and *MP88::V5 mpk1*Δ strains. Equal amounts of protein (3.6 µg) from each sample were separated by SDS-PAGE, transferred to a nylon membrane, and probed with an anti-V5 antibody (R960-25; Invitrogen). Molecular weight markers are indicated in kDa. (**F**) Analysis of EV proteomic data from WT and *mpk1*Δ strains showing the distribution of protein families in the EV proteome. The percentage of iBAQ values corresponding to proteins in each category is shown. Membrane, GPI-anchored, and mitochondrial proteins were identified using DeepTMHMM-1.0 (39), PredGPI (40), and Mitofate (41), respectively. (**G**) Western blot analysis of WT and *mpk1*Δ EVs using an anti-histone H3 (ab8580; Abcam). Equal amounts of EVs (2.9 × 10^10^ EVs) from each sample were loaded in an SDS-PAGE. EV proteins were then transferred to a nylon membrane and probed with an anti-histone H3 antibody. An unpaired, two-tailed *t* test and one-way analysis of variance followed by Dunnett’s multiple-comparison test were used for statistical analysis. Error bars represent the mean ± SD of three biological replicates. *P < 0.05; **P < 0.01.

We reasoned that the reduced surface decoration observed in *mpk1*Δ EVs could be associated with a decreased abundance of proteins responsible for forming these structures in EVs produced by the mutant strain. Consistent with this hypothesis, EV protein extracts from the *mpk1*Δ mutant contained less protein (0.08 µg of proteins/10^8^ EVs ± 0.008) than those from the WT (0.24 µg of proteins/10^8^ EVs ± 0.05; Fig. 5A). Similar results were obtained from the *mkk2*Δ and *bck1*Δ mutants (Fig. 5A).

To validate this observation, we V5 tagged the protein Mp88, which is one of the most abundant proteins associated with EV extract (15). This GPI-anchored protein is also known to be highly mannosylated (42), and it is thought to be a major component of the EV fibrillar decoration in *C. neoformans* (15). Although the precise function of Mp88 remains unclear, we previously showed that an *mp88*Δ mutant strain is more sensitive to SDS and less efficiently phagocytosed by macrophages (43). Because terminal tagging disrupts maturation of GPI-anchored proteins (44), we generated a series of internal V5 insertions within Mp88. Only insertion after Gln125 preserved Mp88 function, as assessed by SDS sensitivity (Fig. 5B). Using this functional allele, we found that Mp88 abundance was markedly reduced in EVs from the *mpk1*Δ mutant despite equal protein loading (Fig. 5C through E). Together, these results suggest that the CWI pathway regulates not only the quantity of proteins associated with EVs but also the composition of the EV proteome.

To further confirm this hypothesis, we performed comparative proteomic analysis on EV protein extracts isolated from *mpk1*Δ and KN99α strains, using three biological replicates (Table S2). Consistent with our previous findings (15) and the results of the western blot presented above, GPI-anchored proteins were among the most abundant components of the wild-type EV proteome, accounting for 22% of the total iBAQ signal (Fig. 5F). In contrast, this category of proteins was markedly reduced in the mutant EV proteome, representing only 2.5% of the total. The EV proteome was extensively remodeled in the mutant strain. For instance, ribosomal proteins accounted for 54.4% of the mutant EV proteome, compared to only 20.2% in wild-type EVs (Fig. 5F) (Table S2). Similarly, we observed that histone proteins were significantly more abundant in the *mpk1*Δ mutant EV proteome compared to the wild type, representing 4.5% and 0.5% of the total EV proteins in each strain, respectively. This observation was confirmed by western blot analysis using an anti-H3 antibody (Fig. 5G). Overall, these results demonstrate that *MPK1* regulates EV protein composition in *C. neoformans*.

### The *mpk1*Δ mutant produces apoptotic-like EVs

**Fig 6:**
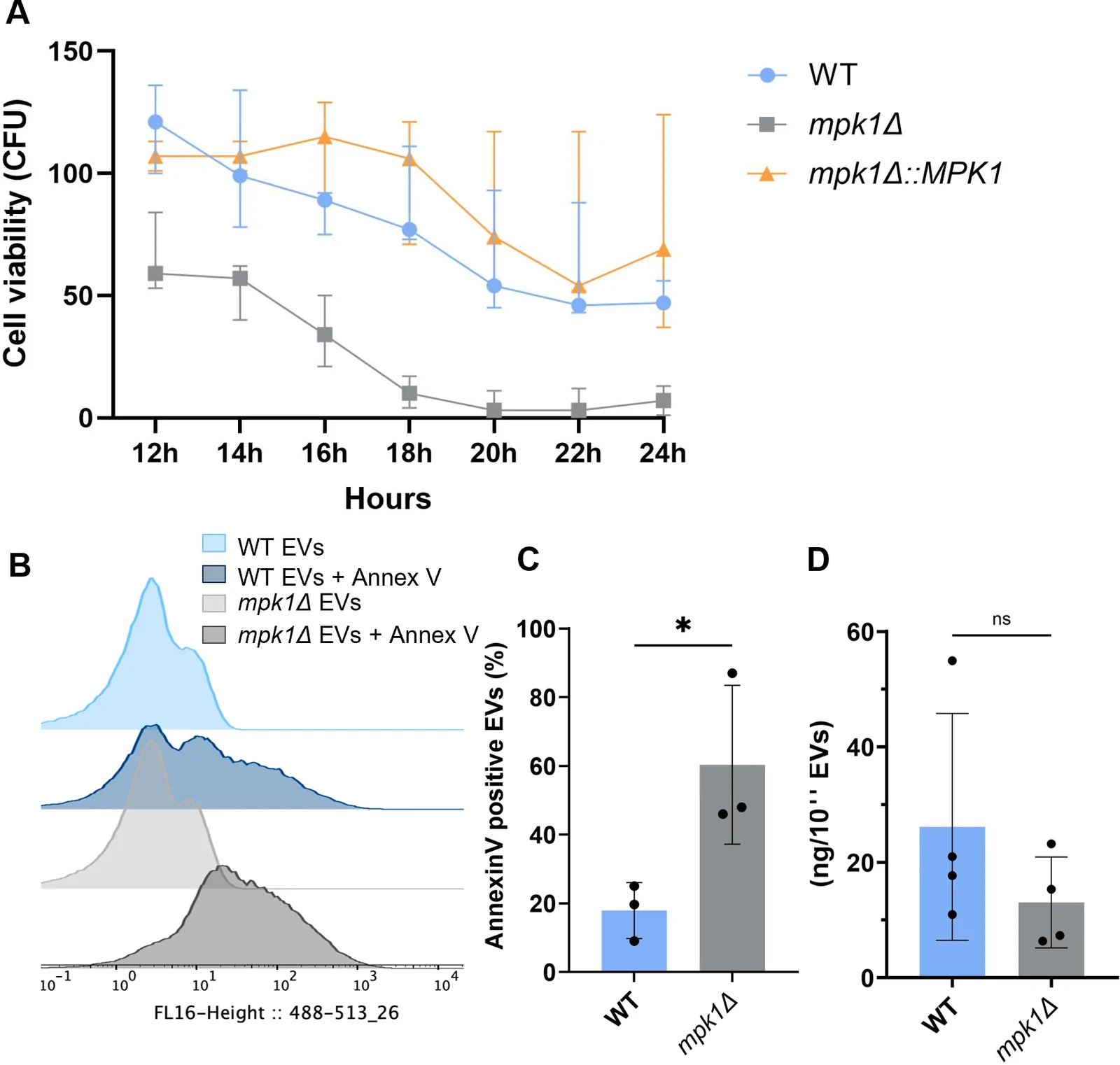
Characteristics of *mpk1*Δ EVs. (**A**) Cell viability over the course of EV production on SD plates. WT, *mpk1*Δ*::MPK1*, and *mpk1*Δ cells were recovered from SD medium after different time points. Adapted dilutions were prepared, and 100 cells were spread on YPD medium. CFUs were counted after 48 h of incubation at 30°C. (**B and C**) Flow cytometry analysis on WT and *mpk1*Δ EVs labeled by annexin V-FITC. Data were acquired using a MoFlo Astrios flow cytometer (Beckman Coulter). Data were analyzed using FlowJo software. (**D**) Quantification of EV DNA content associated with EVs by a Qubit 4 Fluorometer using the Qubit dsDNA HS Assay Kit. Significance was determined by performing an unpaired *t*-test (two-tailed) for DNA content and annexin-V labeling. Error bars show the mean of three biological replicates ± SD. ns, non-significant; *P < 0.05.

The enrichment of ribosomal proteins and histones in *mpk1*Δ EVs resembled the protein composition of mammalian apoptotic EVs (ApoEVs) (45, 46). In mammalian systems, the production of ApoEVs is associated with programmed cell death (47). Consistent with this hypothesis, colony-forming unit (CFU) quantification following plating of appropriate dilutions of cells grown for different durations in synthetic dextrose (SD) medium onto yeast extract-peptone-dextrose (YPD) plates revealed a major loss of viability in the *mpk1*Δ mutant (Fig. 6A). Similar results were obtained with *mkk2*Δ and *bck1*Δ mutants, suggesting a general characteristic of CWI mutant strains (Fig. S3). Another key hallmark of ApoEVs in mammals is the exposure of phosphatidylserine (PS) at their outer membrane (45, 48). During apoptosis, PS—normally confined to the inner leaflet of the plasma membrane—is translocated to the outer leaflet through activation of scramblases and inhibition of flippases (49). This membrane asymmetry can be detected using annexin V, a calcium-dependent protein that binds PS with high affinity (50). Interestingly, we observed a significantly higher proportion of annexin V-positive EVs in the *mpk1*Δ mutant (60.33% ± 23.12%) compared to the wild-type parental strain (17.9% ± 8.15%), indicating that a subset of EVs produced by the *mpk1*Δ mutant display apoptotic-like features (Fig. 6B and C). However, despite the increased abundance of histones in EV protein extracts from the *mpk1*Δ mutant, we did not detect a statistically significant increase in EV-associated DNA content compared to the parental strain (*mpk1*Δ: 13.06 ± 7.88 ng per 10^11^ EVs; WT: 26.14 ± 19.65 ng per 10^11^ EVs), in contrast to what has been reported for mammalian ApoEVs (48, 51) (Fig. 6D). Nevertheless, our data revealed that *C. neoformans* can produce a previously undescribed category of fungal EVs that shares several characteristics with mammalian apoptotic EVs and that the CWI kinase Mpk1 regulates their production.

## DISCUSSION

In this study, we conducted a forward genetics screen of a collection of kinase mutant strains to identify regulators of EV production in *C. neoformans*. Using the same strategy previously applied to a transcription factor mutant library (14), we confirmed the robustness of our screening approach (24). However, unlike our previous screen, which identified only positive regulators of EV production, the present kinase mutant screen also revealed mutants that overproduce EVs. These mutants provide the first genetic evidence that fungal EV production is regulated by both positive and negative signaling pathways. This further supports the idea that EV secretion is an actively controlled process rather than a by-product of growth (14, 52).

Phenotypic analysis of mutant strains altered in EV production confirmed the apparent co-regulation of fluconazole sensitivity and EV production previously reported (14). EVs have been proposed to play a role in regulating drug resistance in fungi (53). For example, EVs can induce amphotericin B resistance in *Candida auris* (54). In *Candida tropicalis*, EVs can suppress the inhibitory effect of caspofungin and fluconazole on biofilm formation (55). EVs have also been shown to be implicated in regulating drug resistance in bacteria. For example, antibiotics can stimulate the release of protein-containing vesicles from a subpopulation of *Escherichia coli* cells, enabling protein exchange with persister cells (56). In fungi, one possible explanation is that mutations affecting EV production also alter plasma membrane lipid homeostasis, thereby influencing susceptibility to azole antifungals. However, the molecular basis linking EV production and fluconazole susceptibility remains unknown.

Our analysis revealed that CWI mutant strains produce more EVs than the wild-type strain. However, the EVs produced by these mutant strains are of a different type. Increasing evidence suggests that fungal EVs comprise heterogeneous populations analogous to those described in mammalian and plant systems, where distinct EV subclasses can be distinguished by specific protein markers and biogenesis pathways (57–59). In fungi, however, molecular markers defining EV subpopulations remain largely absent, with claudin-like Sur7 in *C. albicans* representing one of the few proposed examples (60). Previous observations based on cryo-EM, flow cytometry, proteomic, and genetic analyses have suggested EV heterogeneity in several fungal species (4, 5, 13, 15, 61–63), yet none of these populations have been characterized in detail and directly linked to a biosynthetic pathway.

EVs produced by the *mpk1*Δ strain are mostly undecorated and have a different protein content. Mp88, one of the major EV proteins in the wild type, appears to be nearly absent in the mutant EVs and can be considered an anti-marker of these vesicles. These EVs share several features with mammalian ApoEVs, although whether they represent bona fide fungal ApoEVs remains to be established (64). Like ApoEVs, *mpk1*Δ EVs are larger than wild-type EVs and enriched in histone proteins. However, *mpk1*Δ EVs do not contain more DNA than wild-type EVs, which is reminiscent of some subtypes of mammalian ApoEVs (48, 65). In mammals, histone proteins can be exposed on the surface of EVs under stress and are not necessarily associated with DNA (66). More importantly, they are labeled by annexin V, which is consistent with increased exposure of phosphatidylserine on their outer leaflet. Interestingly, annexin V labeling of wild-type EVs suggests that these Apo-like EVs are also produced by the wild-type strain, albeit at lower concentrations.

Increased production of CWI-associated EVs correlates with elevated cell death, which is consistent with their ApoEV-like properties. Whether these vesicles arise directly from dying cells or instead result from alterations in cell wall remodeling remains unresolved. The CWI pathway is a major regulator of cell wall integrity and remodeling (25, 30, 32), making both possibilities plausible. Conversely, the predominance of decorated EVs in the wild-type strain suggests that most EVs are generated through distinct biogenesis pathways (5). Nevertheless, contributions from other regulated cell death pathways cannot yet be excluded.

The CWI pathway could also provide a mechanism linking environmental stress to EV production. In fungi, cell wall integrity is challenged by a variety of environmental conditions, including osmotic stress, cell wall perturbation, temperature changes, and oxidative stress (25, 30, 32). Such stress signals may therefore modulate EV production by altering cell wall remodeling and the ability of vesicles to traverse the cell envelope. Thus, Mpk1-dependent EV regulation may represent a mechanism by which *C. neoformans* adjusts EV production and cargo composition in response to changes in its extracellular environment. The increased EV production observed in the *mpk1*Δ mutant could consequently reflect a constitutive response to altered cell envelope homeostasis, mimicking aspects of a stress-induced EV response.

The diversity of fungal EVs remains poorly understood and is likely underappreciated. As has been demonstrated in other organisms, improved EV purification approaches, including differential centrifugation, density gradient centrifugation, size-exclusion chromatography, and affinity purification (24, 58), together with the identification of EV markers that are specific to, or enriched in, distinct EV subpopulations, will be essential for resolving this heterogeneity. The *MP88::V5* strain generated here provides the first genetically encoded EV marker in a human fungal pathogen and therefore establishes a valuable platform for studying EV production. In the future, additional EV-associated proteins (9, 15) should be tagged, and/or antibodies against EV proteins should be generated and evaluated for their ability to distinguish specific EV subpopulations. Our findings also confirm the existence of multiple pathways that regulate the production of distinct EV populations. However, only a few components within these pathways have been identified thus far. Advancing our understanding will require complementary strategies, including the detailed characterization of the mutants identified in this study, additional genetic screens, and transcriptomic analyses to identify genes that are differentially regulated during EV production. It will also be important to develop and apply microscopy-based approaches using the EV markers described above. High-resolution microscopic analysis of both EVs and the cells producing them is critical for directly visualizing EV release, determining the cellular origin of distinct EV populations, and linking molecular markers with their spatial and functional contexts.

A recent study demonstrated that fungal EVs are produced during infection *in vivo* (6), providing strong support for their physiological relevance. Nevertheless, the functions of fungal EVs during infection are not well understood. Defining the molecular pathways that control EV production, establishing reliable markers for distinct EV populations, and determining how each EV subtype contributes to fungal physiology and host-pathogen interactions are now major challenges for the field. The genetic resources and EV markers developed in this study provide a framework to address these questions.

## MATERIALS AND METHODS

### Media and culture conditions

*C. neoformans* strains were maintained at −80°C in 40% glycerol and routinely cultured on YPD agar (1% yeast extract, 2% peptone, 2% glucose, and 2% agar). Overnight cultures were grown in liquid YPD at 30°C with shaking (180 rpm). EVs were isolated from cells grown on SD agar (0.67% yeast nitrogen base without amino acids, 2% glucose, and 2% agar). Selective media contained nourseothricin (100 µg/mL) or G418 (200 µg/mL). Capsule induction was performed in induction medium as previously described (67).

### Fungal strains and mutant construction

The *C. neoformans* kinase library (23) was obtained from the Fungal Genetic Stock Center (USA). The *mkk2*Δ mutant was generated by CRISPR-Cas9-mediated homologous recombination using the TRACE system (68). Complemented strains were obtained by integrating the corresponding WT gene, including its native promoter and terminator, into the Safe Haven 1 locus (36). The *MP88*-V5 tagged strain has been constructed by introducing a V5 tag right after Gln125. Correct integration and gene deletion were confirmed by PCR. All strains, plasmids, and primer sequences used in this study are listed in Tables S3 to S5, respectively.

### EV isolation and quantification

EVs were isolated from cells grown on solid SD medium for 22 h at 30°C. Previously published rapid and ultracentrifugation-based protocols were used to prepare EV extracts (24). EV quantification was obtained by measuring the quantity of sterol in the extract using the Amplex Red Cholesterol Assay kit (Invitrogen, A12216) or by nano flow cytometry using a NanoFCM instrument as previously described (24).

### Mutant library screening

The kinase mutant collection (23) contains two independent deletion strains for each kinase gene. We screened this collection for defects in EV production using one deletion strain for each kinase gene, following the rapid screening protocol described previously (24). As described in the Results section, mutants producing less than 25% or more than 1.7-fold the number of EVs produced by the WT strain were selected for further analysis. For each selected gene, both independent deletion mutants were subsequently tested in at least three biological replicates. The values reported in Table 1 represent the mean across all biological replicates for the two independent mutants.

### EV cryo-EM analysis

Cryo-EM analysis was done as previously described (15). EVs from parental strain and *mpk1*Δ mutant were prepared by the conventional ultracentrifugation method, and 4 μL were spotted on glow-discharged lacey grids (S166-3, EMS) and cryo-fixed by plunge freezing at −180°C in liquid ethane using a Leica EMGP (Leica, Austria). Grids were observed with Tecnai F20 (Thermo Fisher Scientific) operating at 200 kV, and images were acquired under low-dose conditions using the software EPU (Thermo Fisher Scientific) and a direct detector Falcon II (Thermo Fisher Scientific). Based on the cryo-EM images obtained, more than 100 EVs were used to measure EV size and observe fibrillar decorations. EV diameters were measured using Fiji/ImageJ software and calculated by taking four different measures of EV diameter, delimitated by the lipid bilayer.

### EV labeling by cytometry

EVs produced by ultracentrifugation were labeled with the Concanavalin A (ConA)-Alexa Fluor 488 conjugated for mannoproteins and with the Alexa 488 labeled anti-GXM monoclonal antibody 18B7 for GXM (69) as previously described (15). For annexin V-FITC labeling, 2.5 µL of annexin V-FITC (BD Biosciences) was added in a 1.5 mL Eppendorf tube containing 100 µL of 0.02 µm-pore filtered binding buffer (HEPES 10 mM pH 7.4, NaCl 140 mM, and CaCl_2_ 0.25 mM) with 1.10^10^ EVs and incubated 15 min at RT, protected from light. After incubation, the samples were transferred to BD Trucount tubes (BD Biosciences) for flow cytometry analysis. EVs were analyzed on a cell sorter MoFlo Astrios (Beckman Coulter) equipped with an EQ module to detect nanoparticles, with 488 nm and 561 nm lasers at 200 mW as previously described (15).

### EV protein quantification and western blot analysis

EV protein content was assessed by Pierce BCA protein assay kit (Thermo Scientific, 23227). For western blots, proteins were separated on 4%–12% Bis-Tris gels (NuPAGE, Invitrogen), transferred to nitrocellulose membranes, and detected using anti-V5 (R960-25, Invitrogen) or anti-histone H3 (ab8580, Abcam) primary antibodies followed by HRP-conjugated secondary antibodies (goat anti-mouse IgG antibody, 12349 or goat anti-rabbit IgG antibody, 12348, Sigma). Signals were visualized using Clarity ECL substrate (1705061, Bio-Rad).

### Proteomic analysis

#### TCA/acetone protein precipitation and protein digestion

Vesicle pellets were resuspended in water with protease inhibitors. Proteins were precipitated by adding one volume of pre-chilled 40% TCA and incubated overnight at 4°C. After centrifugation at 14,000 × *g* for 15 min at 4°C, pellets were washed twice with cold acetone, centrifuged, air-dried, and resuspended in buffer for downstream analysis. Samples were lysed in 5% SDS, 50 mM TEAB (pH 8.5), reduced with 5 mM TCEP (15 min at 55°C), and alkylated with 20 mM iodoacetamide (30 min, RT, dark). After acidification with 2.5% phosphoric acid, six volumes of binding buffer (90% methanol, 100 mM TEAB) were added. Samples were loaded onto S-Trap Micro Spin Columns (ProtiFi, USA), washed, and digested overnight at 37°C with sequencing-grade trypsin (1:10 enzyme:protein). Peptides were eluted in three steps, desalted using the Assay MAP Bravo system with the Peptide Clean-up v3.0 protocol (Agilent), dried, and resuspended in 0.1% formic acid.

Peptides were analyzed on an EASY-nLC 1200 system coupled to a Q Exactive Plus mass spectrometer (Thermo Fisher Scientific). Peptides were separated on a 50 cm PepMap C18 column over a 132 min gradient. MS data were acquired using a data-dependent Top 10 method with survey scans (300–1,700 m/z) at 70,000 resolution and MS/MS scans at 17,500 resolution. Dynamic exclusion and HCD fragmentation (NCE 27) were used.

Raw MS data were processed using MaxQuant v2.0.3.0 (70) and the Andromeda search engine (71), searched against the CNAG protein isoform database (72), known contaminants, and reversed sequences. Trypsin specificity was used with two missed cleavages. Modifications included carbamidomethylation (Cys, fixed), oxidation (Met), and N-terminal acetylation (variable). False discovery rate (FDR) was set at 1%. Quantification was performed with LFQ (Fast mode [73]). Protein abundance was also estimated using iBAQ intensity values (74). After filtering, intensities were log2-transformed, normalized (75), and missing values imputed (imp4p) using the impute.mle function of the imp4p R package (76), when at least one observed value was present in that condition. This algorithm imputes values in a condition only when an intensity value has been quantified in at least one of the samples of the considered condition.

### Evaluation of chitin cell wall level

Chitin cell wall level was assessed with Calcofluor White (Fluorescent Brightener 28, Merck; 250 mg/mL) staining and quantified by flow cytometry using the MACSQuant Analyzer (Miltenyi Biotec) as previously described (77). For each sample, a total of 50,000 events were counted. The mean intensity of fluorescence and the standard deviation from the stained and the non-stained population were quantified on FlowJo software (version 10.10), and a staining index was calculated for each strain.

### Cell viability

Cell viability was assessed in the same condition as we produce EVs. From a cell inoculum on YPD, 300 μL of 3.5 × 10^7^ cells/mL were grown on solid SD medium. At each time point, cells were recovered, counted in a particle counter (Coulter Z1, Beckman Coulter), and 100 μL of 10^3^ cells/mL were spread onto YPD solid medium. After 48 h of incubation at 30°C, CFUs were evaluated by counting the colonies on the plate.

### Statistical analysis

Statistical analyses were performed using GraphPad Prism 9 (GraphPad Software). The statistical tests used are indicated in the corresponding figure legends. *P* values < 0.05 were considered statistically significant.

## Data Availability

The mass spectrometry proteomics data have been deposited to the ProteomeXchange Consortium via the PRIDE (78) partner repository with the data set identifier PXD083999.

## SUPPLEMENTAL MATERIAL

Supplemental figures mbio.02302-26-s0001.pdf Fig. S1 to S3.

Table S1 mbio.02302-26-s0002.xlsx Production of EVs by each kinase mutant.

Table S2 mbio.02302-26-s0003.xlsx EV proteome in WT and *mpk1*Δ mutant strain.

Table S3 mbio.02302-26-s0004.xlsx Strains used in this study.

Table S4 mbio.02302-26-s0005.xlsx Plasmids.

Table S5 mbio.02302-26-s0006.xlsx Primers.

ASM does not own the copyrights to Supplemental Material that may be linked to, or accessed through, an article. The authors have granted ASM a non-exclusive, world-wide license to publish the Supplemental Material files. Please contact the corresponding author directly for reuse.
