# Supplementary material for: The cell wall integrity pathway kinase Mpk1 regulates extracellular vesicle production and apoptotic-like vesicle release in Cryptococcus neoformans
Source: mBio. 2026 Oct 23;17(11):e02302-26. doi: 10.1128/mbio.02302-26 (PMC13753817; doi:10.1128/mbio.02302-26)
Supplement: Supplementary file 1 [file mbio.02302-26-s0001.pdf]

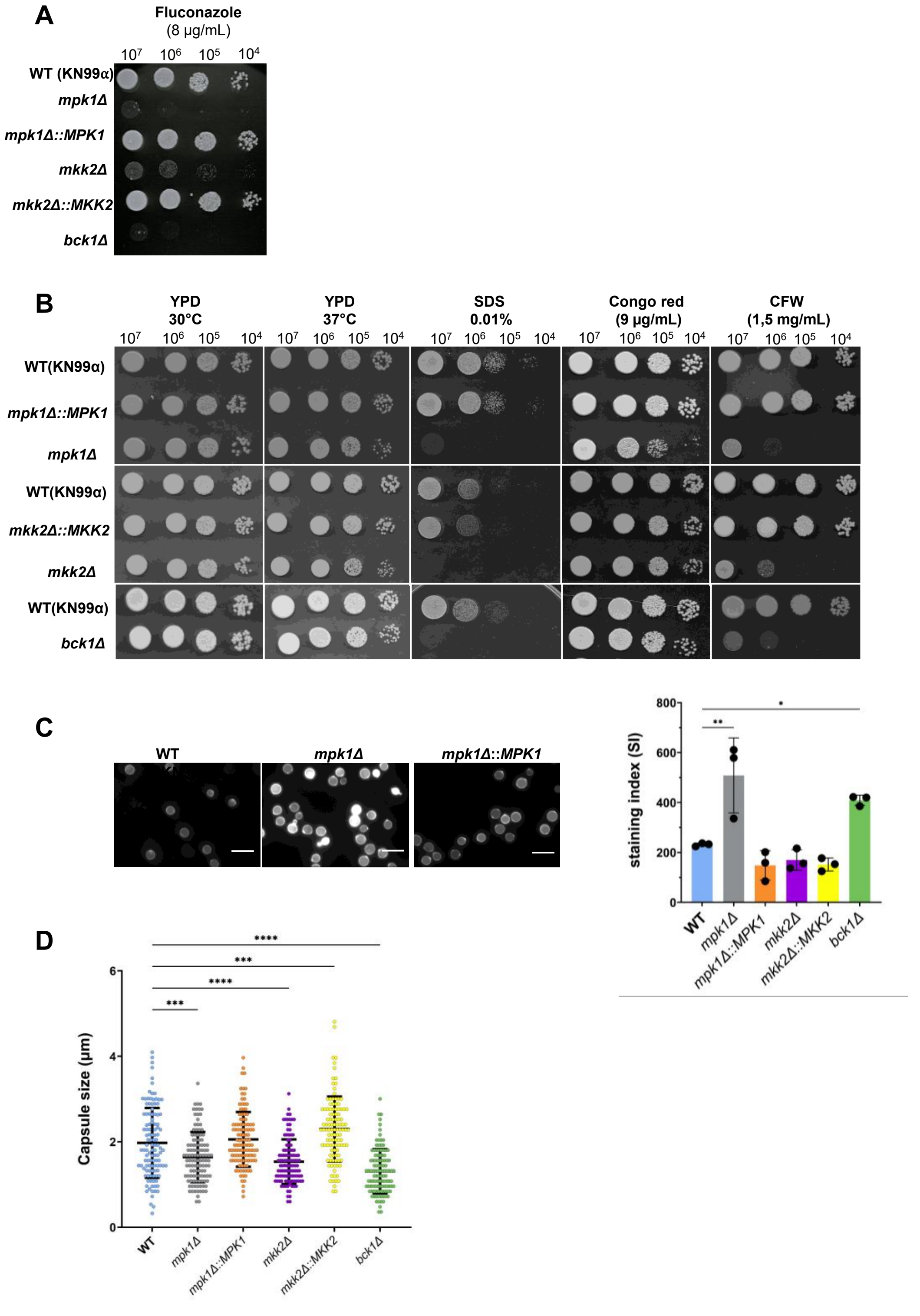

Supplementary figure S1: Phenotypes associated with CWI gene deletion in *C. neoformans* KN99α background.

(A)(B) Fluconazole Temperature, SDS, Congo red and Calcofluor White susceptibility assays. Serial dilutions of CWI mutants (*bck1Δ*, *mkk2Δ*, and *mpk1Δ*) and complemented strains (*mkk2Δ::MKK2* and *mpk1Δ::MPK1*) were spotted on medium containing fluconazole. Plates were incubated for 48 h at 30°C prior to imaging.

(C) Calcofluor White staining (10 µg/mL) of CWI mutants and complemented strains after 16 h of growth on YPD medium. Staining was quantified by flow cytometry using a MACSQuant Analyzer (Miltenyi Biotec), and a staining index was calculated. Scale bars represent 12.5 µm. Representative images are shown.

(D) CWI regulates capsule size in *C. neoformans* KN99α background. Capsule sizes (n=114 per strain) were measured after Indian ink negative staining.

Statistical analyses were performed using one-way ANOVA followed by Dunnett's multiple-comparisons test. Error bars represent the mean ± SD of three biological replicates. *$p<0.05$, ***$p<0.001$, ****$p<0.0001$.

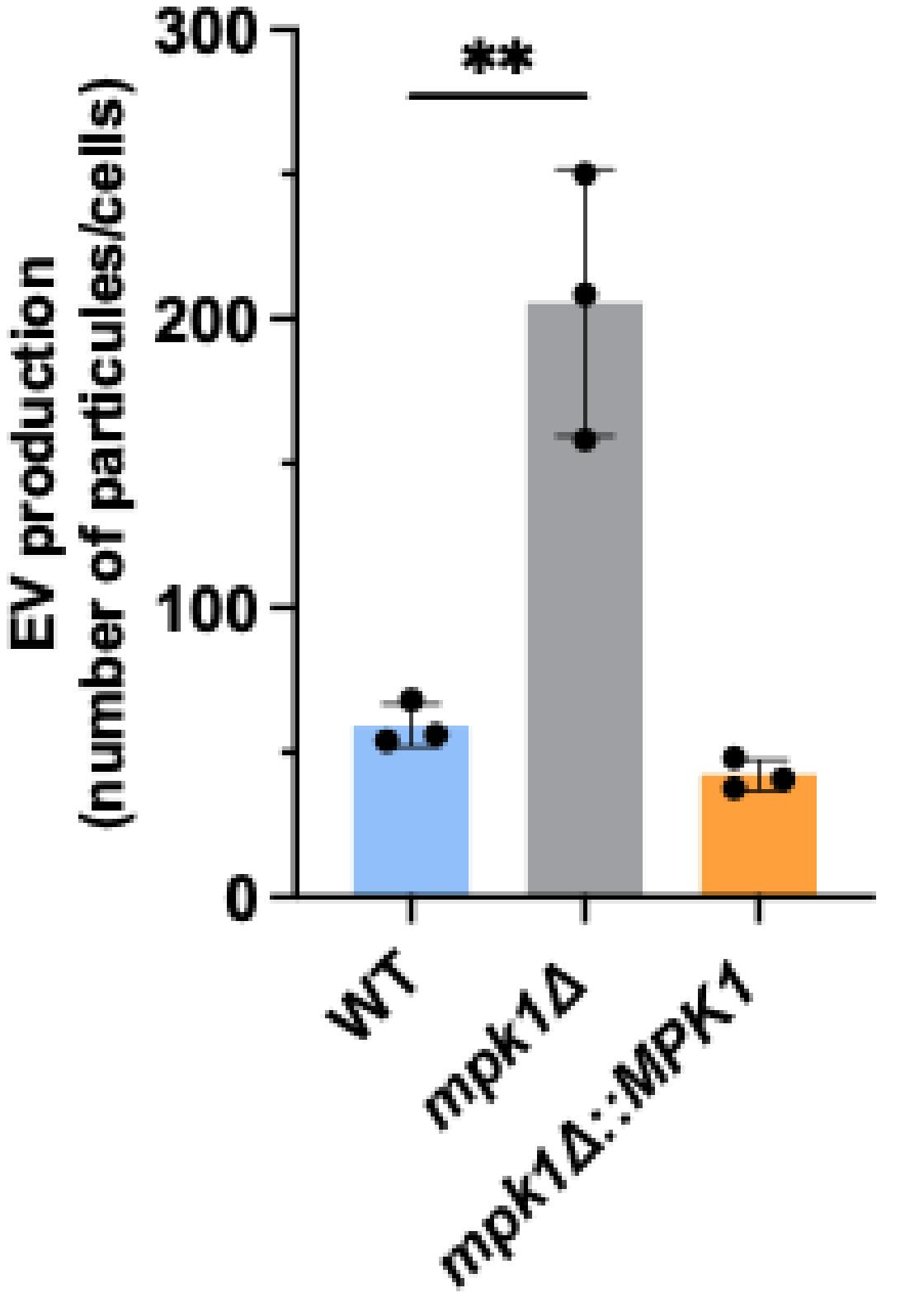

Supplementary figure S2. EV production of WT, *mpk1Δ* and *mpk1Δ::MPK1* strains as evaluated by NanoFCM. EV extracts were prepared using an ultracentrifugation based protocol. Unpaired T-test (two tailed) or one-way ANOVA with subsequent Dunnett's multiple comparisons test have been performed for the statistical analysis. Error bars show mean of three or four biological replicates ± SD. **p<0,01.

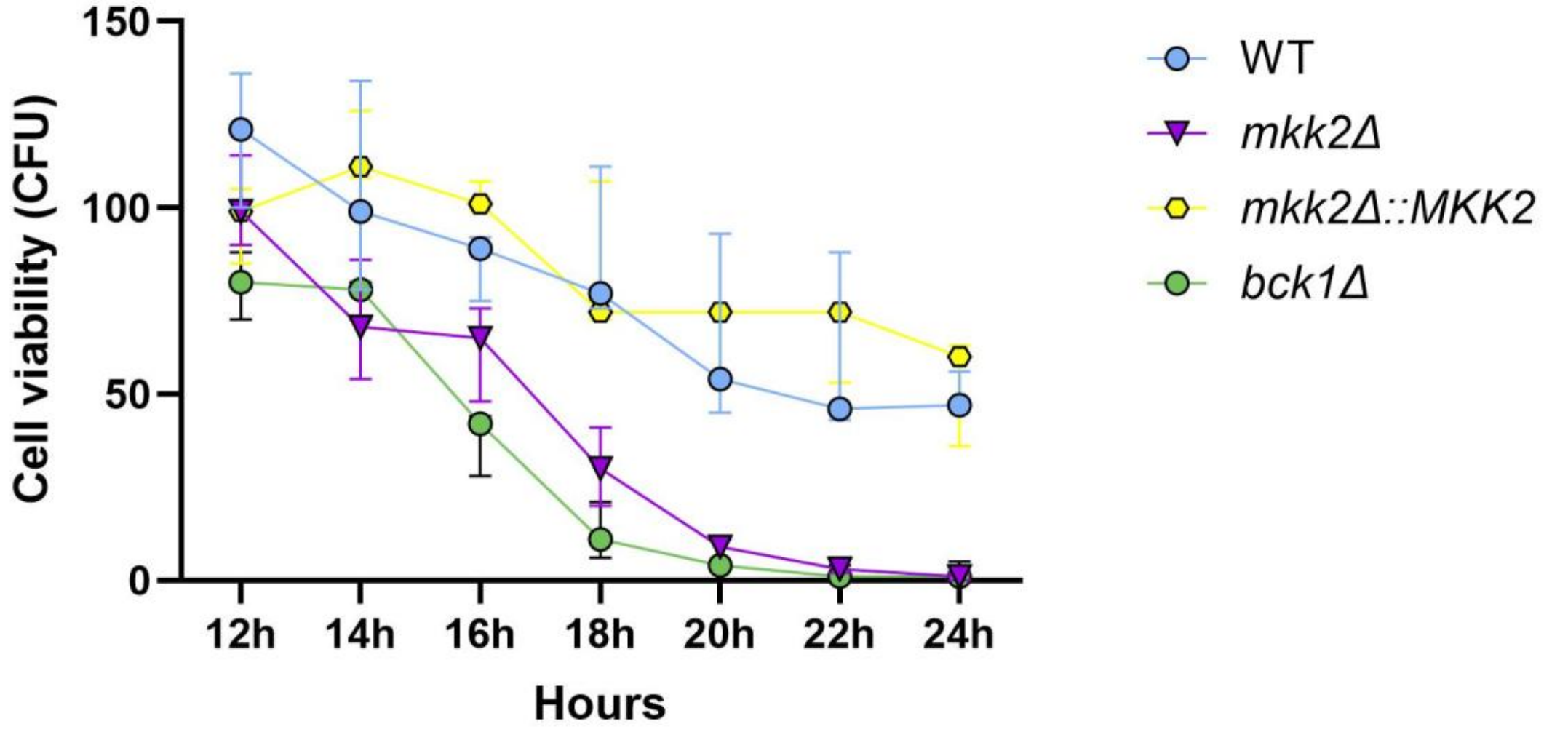

Supplementary figure S3. Cell viability of the *mkk2Δ* and *bck1Δ* deletion mutants. WT, *mkk2Δ mkk2Δ::MKK2* and *bck1Δ* cells were recovered from SD medium after different time points. Adapted dilutions were prepared and 100 cells were spread on YPD medium. CFUs were counted after 48h of incubation at 30°C.
